# Dynamic Hip Screw Fixation of a Proximal Femur Fracture in a Patient With Ipsilateral Above-Knee and Contralateral Hindquarter Amputations

**DOI:** 10.7759/cureus.99246

**Published:** 2025-12-15

**Authors:** Arash Ghoroghi, Muhammad Sohail, Vedran Curkovic, Anil Singhal

**Affiliations:** 1 Orthopaedics, Prince Charles Hospital, Merthyr Tydfil, GBR

**Keywords:** above-knee amputation, bilateral lower limb amputation, dynamic hip screw (dhs), femur intertrochanteric fracture, hemipelvectomy

## Abstract

Intertrochanteric hip fractures are usually managed with dynamic hip screw (DHS) fixation, but standard positioning and traction are difficult in patients with major lower-limb amputations. We describe a 77-year-old woman with a right above-knee amputation and no contralateral limb who sustained an intertrochanteric fracture after a wheelchair fall. The main challenge was achieving axial traction and rotational control on a very short femoral stump without damaging it or compromising imaging. We achieved closed reduction by inserting a distal femoral Steinmann pin and attaching a stirrup to apply controlled traction and rotation on the fracture table, followed by standard DHS fixation through a lateral approach. The patient returned to her pre-injury level of bed-to-wheelchair transfers, and this case outlines a reproducible positioning and traction strategy for similar complex presentations. This case highlights a reproducible skeletal traction strategy that provides safe imaging corridors and controlled reduction even in the absence of the contralateral limb.

## Introduction

Hip fractures are a major concern in older adults, causing functional decline, loss of independence, excess short-term and one-year mortality, and a healthcare and social care burden [1-4]. The hospital costs attributable to incident hip fractures in the United Kingdom are estimated at over £800 million per year and exceed £1 billion when total hospital resource use is considered [2]. Dynamic hip screw (DHS) fixation is an established method for managing stable extracapsular intertrochanteric fractures (e.g., AO/OTA 31-A1 and selected A2 patterns), providing controlled collapse to encourage union, and is widely used in older patients [5,6]. DHS consists of a large lag screw inserted into the femoral head and neck, connected to a lateral side plate, allowing dynamic axial compression across the fracture with progressive loading [6].

Standard technique involves closed reduction under fluoroscopy on a fracture table, longitudinal traction via a boot or foot holder on the injured limb, controlled internal rotation to restore alignment, and counter-traction through a padded perineal post, while the contralateral limb is positioned to permit anteroposterior and lateral image intensifier views [7,8]. Correct positioning is critical because malreduction, poor fluoroscopic access, or excessive soft-tissue pressure can prolong operative time and increase the risk of mechanical failure [6-8].

These steps are challenging in patients with major lower-limb amputations. In above-knee or bilateral above-knee amputees, there may be no distal segment to engage a traction boot, the stump may be too short or conical to tolerate skin traction, and counter-traction and rotational control may be unreliable, jeopardizing reduction and intraoperative imaging [7-12]. The authors have described improvised solutions, including adhesive taping of the stump to the traction arm, skeletal traction using a Steinmann or Denham pin in the distal femoral stump, and external distractors or modified positioning, but guidance remains sparse and heterogeneous [7-12]. This report describes DHS fixation of an intertrochanteric fracture in a patient with an ipsilateral short transfemoral stump and a contralateral hindquarter amputation, a constellation that eliminates the usual contralateral limb for counter-traction and further complicates positioning and fluoroscopic access.

## Case presentation

**Figure 1:**
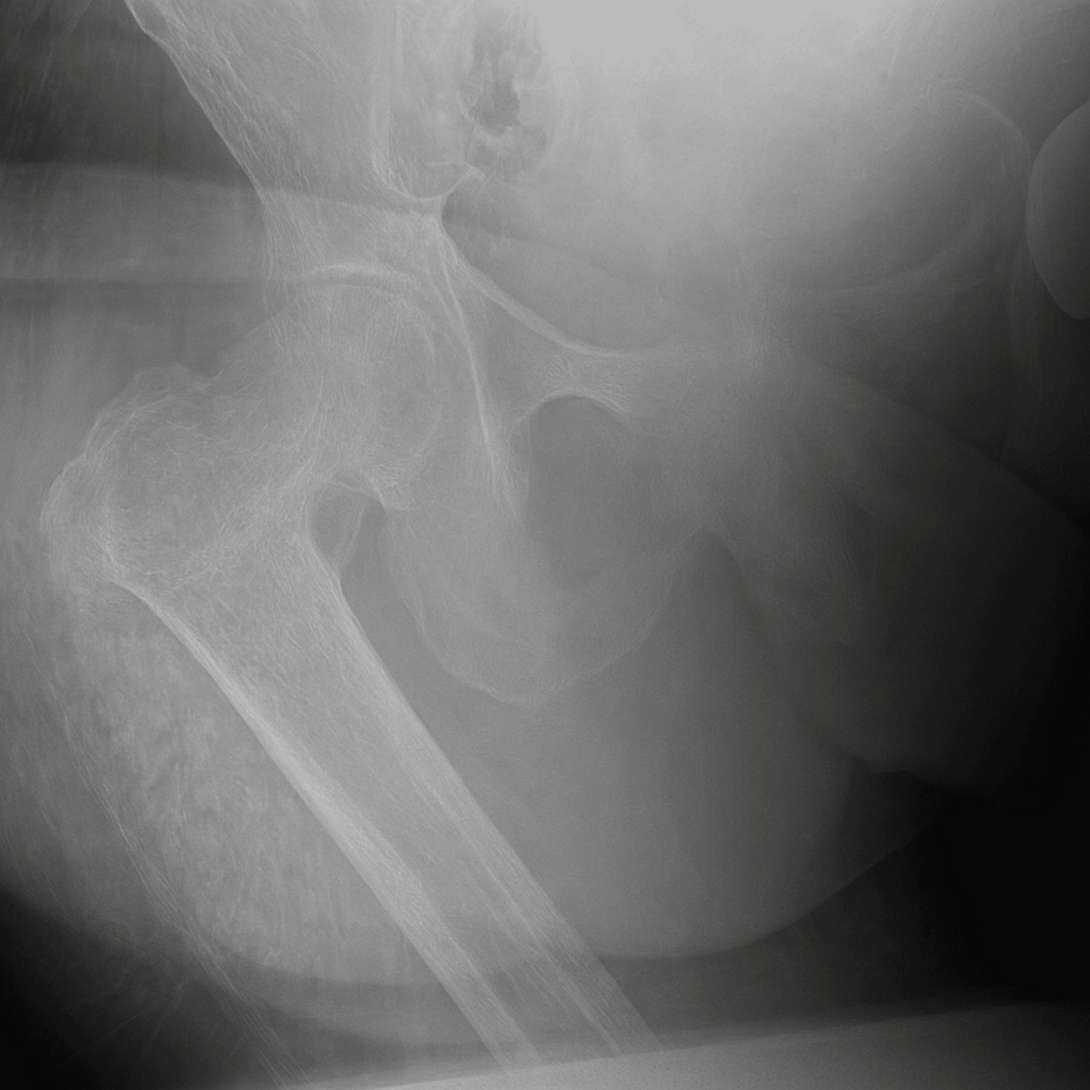
Preoperative radiograph showing a comminuted intertrochanteric fracture

**Figure 2:**
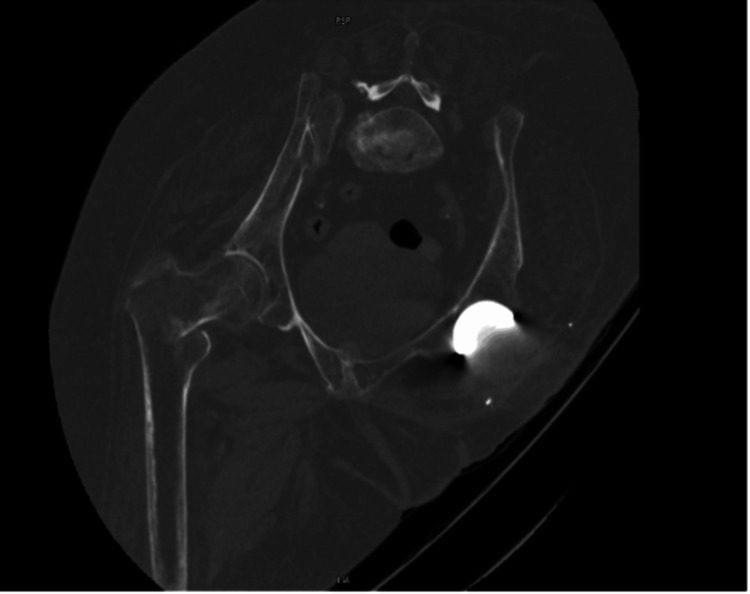
Preoperative CT showing a comminuted intertrochanteric fracture

A 77-year-old wheelchair-bound female patient presented to the emergency department following a fall out of her wheelchair, sustaining a right-sided hip injury. She had a right above-knee amputation and left hindquarter amputation 12 years prior to this presentation, following a road traffic accident. These were carried out as life-saving procedures in staged operations. Plain radiographs revealed a comminuted intertrochanteric fracture of the right neck of the femur. This was further examined with a CT scan of the hip with 3D reconstruction (Figures 1, 2).

**Figure 3:**
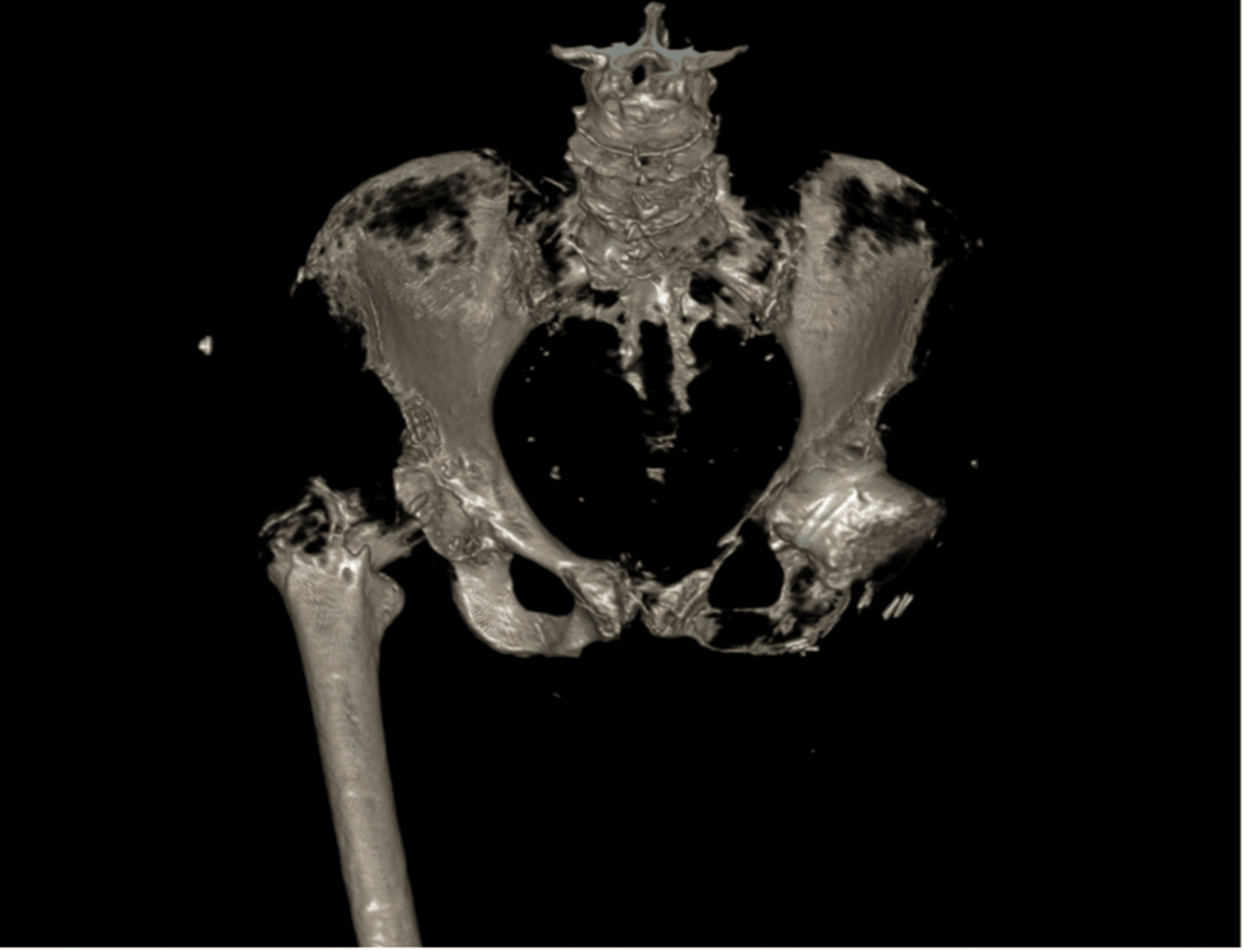
3D CT reconstruction of the right hip fracture

The initial investigation of the patient was complicated by the logistical challenges faced in transferring and orienting her for imaging. The interpretation of the images was assisted by a 3D CT reconstruction (Figure 3). As an independent amputee who was self-transferring onto an electric wheelchair, the patient was planned for fixation of the right intertrochanteric fracture with general anesthetic and fascial block.

**Figure 4:**
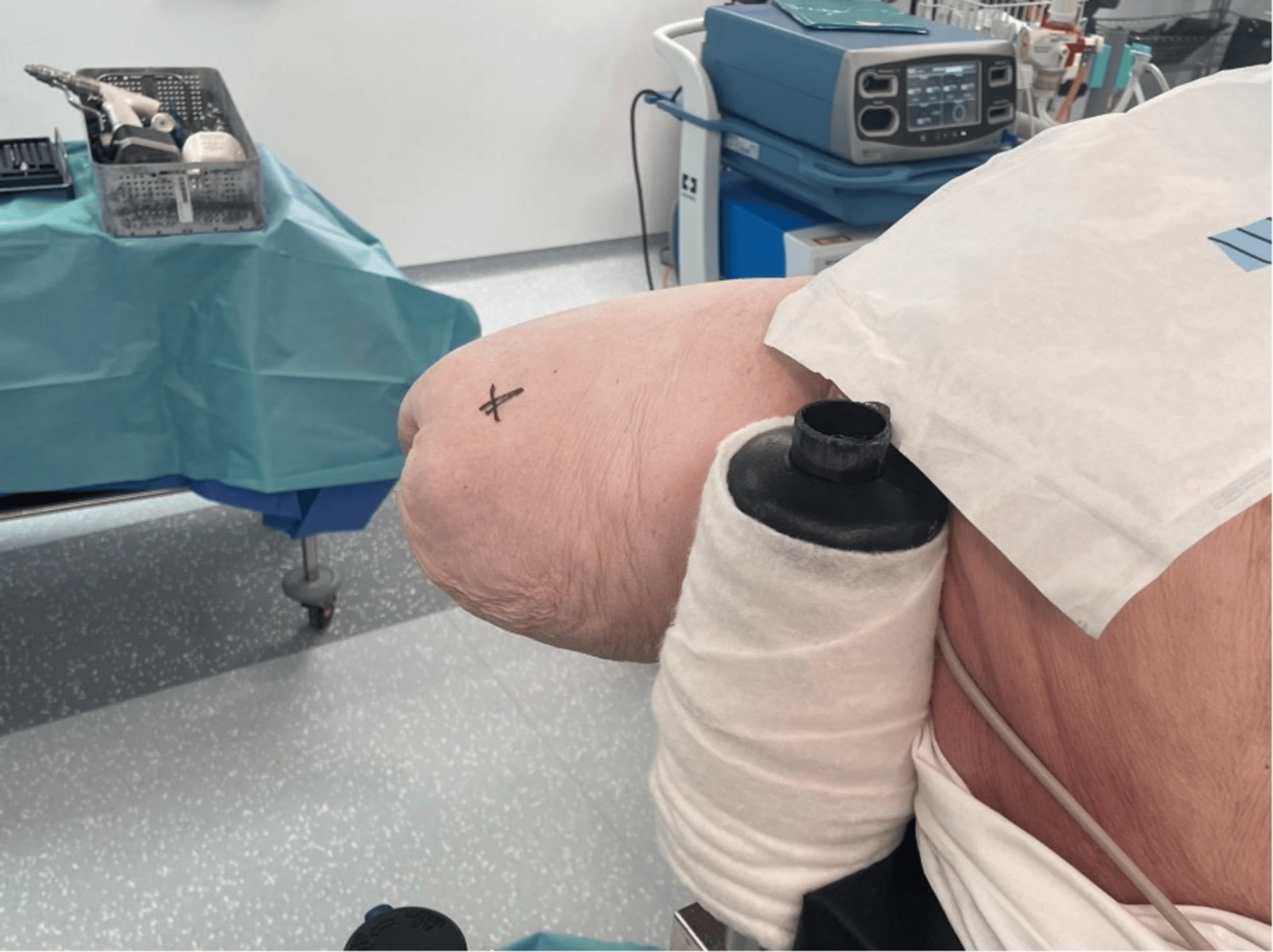
Clinical photograph of the short, conical femoral stump

The major challenge in this case was the positioning of the patient on the traction table and achieving reduction, given the prior amputations. In this case-, the patient was positioned supine with a padded perineal post. Adhesive fabric tape could not be used for skin traction due to the skin health and concerns over degloving of the right stump. The skin was intact but extremely friable. Furthermore, the stump was quite short (29 cm long from the ASIS to the stump end) and conical in shape, which made skin traction more difficult (Figure 4). 

**Figure 5:**
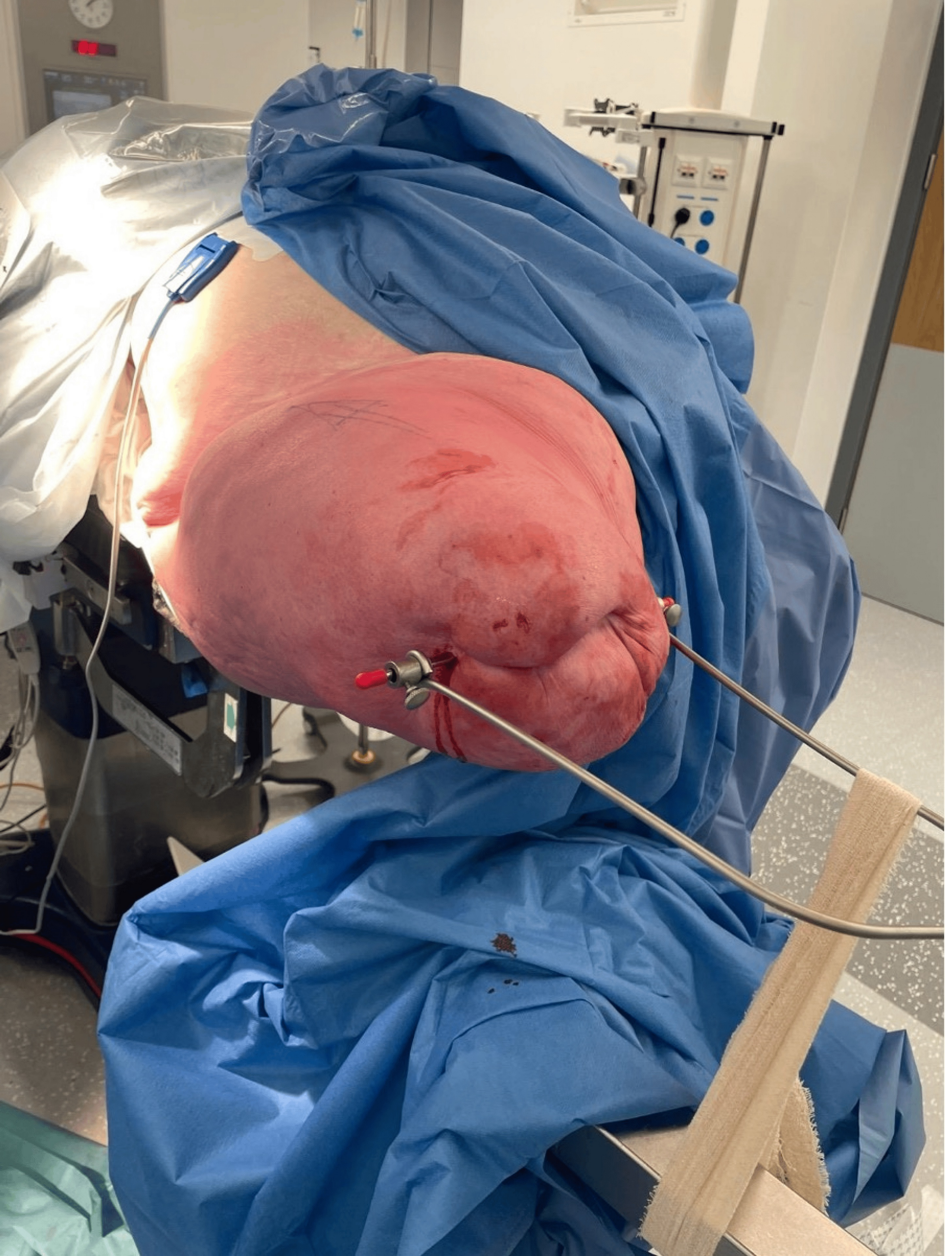
Intraoperative view of Steinman pin placement with Böhler stirrup

**Figure 6:**
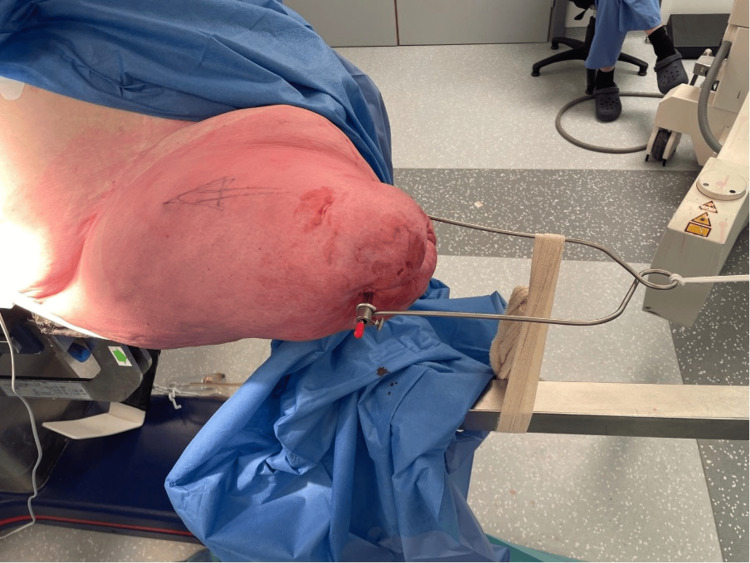
Setup showing attachment of the stirrup to the traction table

In this case, a Steinman pin was placed 5 cm from the distal femoral stump with good hold. Perineal post was used as a standard, but strapping was required to keep the patient’s torso from rotating around the post while maintaining traction. A Böhler stirrup was attached to the Steinman pin, which allowed for internal rotation by fastening to the traction table (Figures 5, 6).

**Figure 7:**
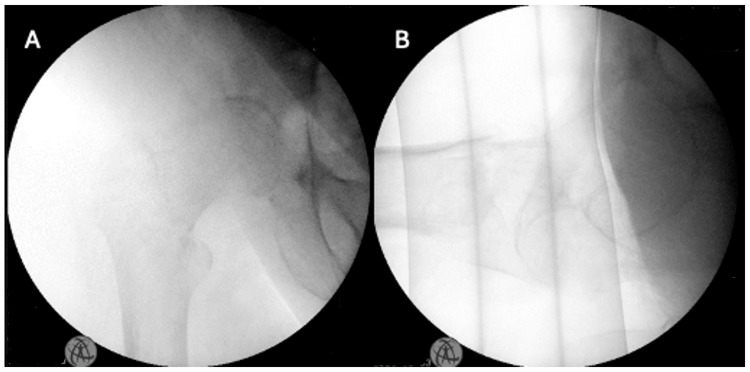
Intraoperative image intensifier confirmation of fracture reduction (A) Antero-posterior view. (B) Lateral view.

Traction was achieved and confirmed by an image intensifier (Figure 7). 

**Figure 8:**
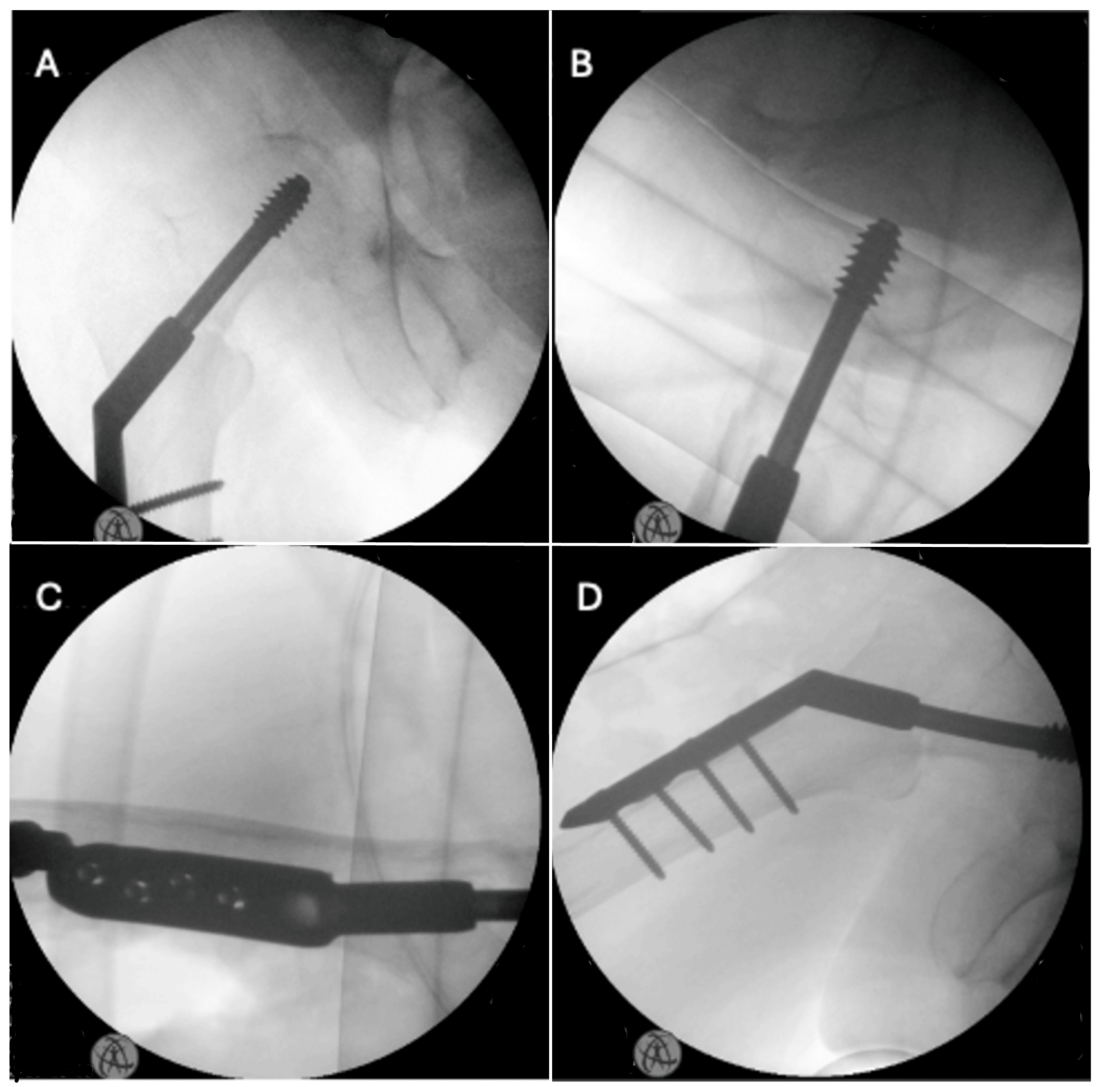
Intraoperative images of DHS insertion (A) Anteroposterior view. (B) Lateral view. (C) Lateral view. (D) Anteroposterior view.

DHS fixation was then carried out as standard with a direct lateral approach. A 95 mm femoral screw and four-hole DHS plate with cortical screws were used (Figure 8). Intraoperatively, it was noted that the bone was soft and there was little feedback from the insertion of the screw. This is in keeping with the patient having osteoporosis. Upon closure and dressing of the lateral incision, the distal femoral pin was removed, and the wound was closed. Postoperative care of the patient included immediate return to transferring from the bed to an electric wheelchair, prior to this admission.

## Discussion

Neck of femur/intertrochanteric fractures in patients with ipsilateral transfemoral amputation are rare, and when combined with contralateral hindquarter amputation, they present an extreme positioning problem: there is no distal limb segment for traction on the injured side and no contralateral limb to provide counter-traction or stabilization against the perineal post. Published studies confirm that intertrochanteric fixation in amputees is technically demanding because routine fracture-table setups assume an intact foot and distal lever arm for traction and controlled internal rotation [7-12]. Inadequate control of axial length, rotation, and abduction/adduction during guidewire insertion and lag-screw placement can compromise reduction and later fixation stability [6-8,10-12].

Several strategies have been reported for similar, but not identical, situations. Aqil et al. described a wheelchair-bound patient with bilateral above-knee amputations in whom DHS fixation was possible without applying traction because the fracture was only minimally displaced; positioning relied on supporting each stump on fracture-table thigh supports and securing them to permit fluoroscopic access [7]. Davarinos et al. reported an ipsilateral extracapsular hip fracture in a unilateral above-knee amputee; they achieved reduction using adhesive fabric tape applied circumferentially to the stump, connected to the traction arm to generate longitudinal pull and internal rotation [8]. That technique depends on adequate stump length and skin tolerance, which were not present in the current case.

Other authors have proposed skeletal traction through the stump. Takeba et al. inserted a Kirschner wire into the distal femoral stump of an above-knee amputee and attached it to the traction rope of the fracture table to achieve controlled longitudinal distraction and partial rotational control during fixation of a trochanteric fracture [9]. Lee et al. described two cases (one above-knee, one below-knee amputation) in which skeletal fixation to the stump permitted intraoperative manipulation on a fracture table, facilitating intertrochanteric fracture reduction and fixation [10]. Patwardhan et al. similarly used a Denham/Steinmann-style pin through the residual femur of an above-knee amputee to connect directly to the traction table, achieving stable reduction for DHS fixation and emphasizing that skeletal traction through bone rather than skin can offer reliable control while preserving fluoroscopic windows [12].

When conventional table traction cannot be applied safely, some teams have abandoned the fracture table entirely in favor of adjunct external devices. Nardulli and Issack reported open reduction and internal fixation of an intertrochanteric fracture proximal to an above-knee amputation using two AO femoral distractors to generate and maintain length and alignment without relying on standard traction boots [11]. This technique can restore axial length and correct varus but requires additional hardware around the proximal femur, which may partially obstruct the C-arm corridor during lag screw or plate placement [11].

Our case differs from most published descriptions in three important ways. First, the ipsilateral stump was short, conical, and at risk of soft-tissue injury, making adhesive skin traction hazardous, unlike the longer, more robust stumps described by Davarinos et al. [8]. Second, the contralateral limb, and thus the usual counter-traction point, was absent because of a prior hindquarter amputation, whereas most reports [7,9,10,12] involved either unilateral amputees with an intact contralateral limb or bilateral above-knee amputees who could still be positioned bilaterally on the table. Third, because the patient’s pre-injury functional baseline was independent transfer between the bed and electric wheelchair, immediate restoration of a stable construct with minimal additional morbidity was a priority, in line with the general principle that early operative fixation and rapid return to pre-fracture mobility status improve functional outcomes and survival after hip fracture [1,3,4].

The solution we used (percutaneous placement of a Steinmann pin approximately 5 cm proximal to the distal end of the femoral stump, attachment of a Böhler stirrup to apply longitudinal traction and internally rotate the stump, strapping of the torso to prevent rotation around a padded perineal post, and subsequent standard DHS insertion) aligns conceptually with previously described skeletal traction techniques [9,10,12] but adapts them to a scenario where there is no contralateral extremity to stabilize against the post. Maintaining the pin distal in the femoral stump provided a bony purchase point for axial traction and rotational control without relying on compromised stump skin, consistent with the wider use of temporary Steinmann pin fixation to aid reduction and stabilize proximal femoral surgery [13]. Importantly, once reduction was confirmed fluoroscopically, DHS placement proceeded through a conventional lateral approach without obstructing the imaging corridor; the pin was removed before closure, leaving no additional hardware in the distal stump that might interfere with prosthetic seating or create focal pressure points, issues which are known to predispose transfemoral amputees to soft-tissue breakdown and hip-flexion contracture if positioning loads are not well-distributed [12,14].

Steinmann pin usage is however associated with risks, including pin-tract infection, iatrogenic fracture through the cortical defect, and occasional deep sepsis or compartment syndrome. Pin-site infection is the most common complication of external fixation, with systematic reviews of pin-site care reporting infection rates between roughly 10% and 100% and highlighting that unchecked infection can progress to osteomyelitis [15]. Compared with skin traction and adhesive-taping methods described for above-knee and bilateral above-knee amputees, Steinmann pin traction has modest requirements for stump length because it relies on distal femoral bone stock rather than a long cylindrical soft-tissue envelope. This allows secure purchase even in very short, conical transfemoral stumps, such as the present case, where a pin 5 cm from the stump tip provided effective traction.

C-arm access is generally favorable with a pin-Böhler stirrup construct, which keeps the stump off the table and preserves anteroposterior and lateral views, whereas some support-based techniques require bulky pads or frames under the stump that can compromise lateral imaging. Rotational control is typically superior with a rigid pin-stirrup linkage, which permits fine, reproducible adjustment of internal rotation throughout guidewire insertion and screw placement compared with adhesive taping or manual support, where rotation is more prone to drift during reaming and implant insertion.

On this basis, we suggest that distal femoral skeletal traction using a Steinmann pin and stirrup is a practical and reproducible method for achieving controlled axial traction and rotational alignment in proximal femoral fracture fixation for patients with short transfemoral stumps, even in the absence of a contralateral limb for counter-traction. This technique offers predictable intraoperative control, preserves fluoroscopic access for guidewire placement and lag screw insertion, and can be discontinued at closure without leaving additional implants in the stump [9-12,14]. Although our follow-up was limited to the immediate postoperative period, the patient rapidly returned to her baseline transfer ability, which is a key functional goal in frail, high-risk hip fracture populations [1,3,4]. Longer-term surveillance would still be necessary to assess union, implant position, stump comfort, and longer-term sitting/transferring tolerance in the wheelchair, but this case supports skeletal traction via a distal femoral pin as a standardizable option when skin traction is unsafe and no contralateral limb exists to provide counter-traction.

## Conclusions

This case highlights a reproducible skeletal traction strategy that provides safe imaging corridors and controlled reduction even in the absence of the contralateral limb. A percutaneous distal femoral Steinmann pin coupled to a Böhler stirrup provided reliable longitudinal traction and rotational control without jeopardizing soft tissue integrity or impeding fluoroscopic access, enabling closed reduction and standard DHS implantation. The patient returned to her pre-injury transfer baseline, underscoring the practicality of this positioning and traction strategy in highly atypical anatomy. 
